# Thoracic Endovascular Aortic Repair Using Stent Grafts in Japan

**DOI:** 10.70352/scrj.ed.25-2001

**Published:** 2025-02-05

**Authors:** Akihiko Usui, Rena Usui, Shunsuke Nakata

**Affiliations:** Cardiovascular Surgery, Fujita Health University, Okazaki Medical Center, Okazaki, Aichi, Japan

**Keywords:** stent graft, thoracic endovascular aortic repair, aortic surgery, number of surgery, mortality

## Abstract

A stent-graft technique was developed by Parodi et al. and has been used clinically for thoracic endovascular aortic repair (TEVAR) since the 1990s. We evaluated how the new stent-graft technology contributed to expanding aortic surgery and improving surgical outcomes of aortic surgery. TEVAR was performed in a limited number of institutes in the early 2000s and was greatly enhanced by the approval of commercially available stent grafts in 2008. Its performance increased steadily thereafter, with 0 cases performed in 1999, 1658 in 2009, and 6461 in 2019. The ratio of TEVAR was 0% in 1999, which increased to 13.9% in 2009 and 28.5% in 2019, respectively. TEVAR has greatly contributed to the improvement of surgical outcomes, especially in non-dissection ruptured aneurysms and type B acute aortic dissection. TEVAR was performed in 53% of ruptured aneurysms, and the 30-day mortality rate improved to 13.9% in 2019 due to a 30-day mortality rate of 12.5% in TEVAR. The effect of TEVAR was more remarkable in patients with acute type B aortic dissection, where the 30-day mortality rate was 5.7%, and the procedure was performed in 75% of cases. The overall 30-day mortality rate improved to 7.0% for all patients with type B acute aortic dissection in 2019. The expansion of TEVAR using stent grafts greatly increased the number of aortic surgeries and played a significant role in improving surgical outcomes. Stent-graft technology has influenced the field of aortic surgery.

## Abbreviations

JATS
: The Japanese Association of Thoracic Surgery
TEVAR
: thoracic endovascular aortic repair

## THE “S” CURVE THEORY

The goal of surgery is to improve a patient’s outcome. To achieve this, surgeons have attempted to develop new surgical techniques. When a novel technique is developed, developers evaluate its early outcomes, identify associated problems, and seek further improvement. We believe that “surgery” is a process of the repeated evaluation and improvement of techniques in pursuit of the best procedure for patients.

A new surgical technique is adopted by pioneering surgeons in the early phase. When related problems are solved and their outcomes stabilize, a new technique is accepted by the majority of surgeons as a common procedure. The adoption rate generally shows an “S” curve form, so it is called “the ‘S’ curve theory” in marketing theory.^1)^

The adoption of a new surgical technique depends on both the invasiveness and the cure rate. The less invasive the technique, the higher its prevalence and acceptance as a common technique, even if the cure rate is relatively low. With the advent of a new technique, the implementation rate of conventional surgery has generally decreased, but a new technique is not applicable to all cases because of anatomical or age-related limitations. Therefore, it is common for conventional techniques to continue to be performed at a certain rate.

I have been involved in aortic surgery for the last three decades, and major new surgical techniques have been introduced in this field. The principal procedure in aortic surgery is open surgery, in which the diseased part of the aorta is replaced with a synthetic graft. However, in Argentina, Parodi et al. developed a stent-graft technique in which a synthetic graft with spring-like expansion was placed inside the aorta to cover the diseased range.^2)^ It has been used clinically for thoracic endovascular aortic repair (TEVAR) since the 1990s.

A stent graft can cause occlusion of the branches at the site of placement. Therefore, the ascending aorta, aortic arch, and upper abdominal aorta, which have major branches, are poor candidates for stent grafting. In addition, endo-leaks may occur during remote periods. The long-term results remain a concern in TEVAR. However, stent grafts are widely used because of their lower invasiveness than open surgery, while TEVAR has anatomical and age-related limitations.

In Japan, hand-made stent grafts were used on a trial basis in limited facilities from the late 1990s to the early 2000s, but commercially available stent grafts for thoracic aortic aneurysms were approved in 2008, and stent grafts for aortic dissections were approved in 2015. Stent-graft technology has since become widespread. The Japanese Association of Thoracic Surgery (JATS) conducted annual surgical surveys from 1997 to 2019, allowing the observation of trends in aortic surgery in Japan over the past 20 years.^3,4)^ We therefore evaluated how the new stent-graft technology contributed to expanding aortic surgery and improving surgical outcomes of aortic surgery and verified the “S” curve theory in the field of aortic surgery.

## AORTIC SURGERY IN JAPAN

**Fig. 1:**
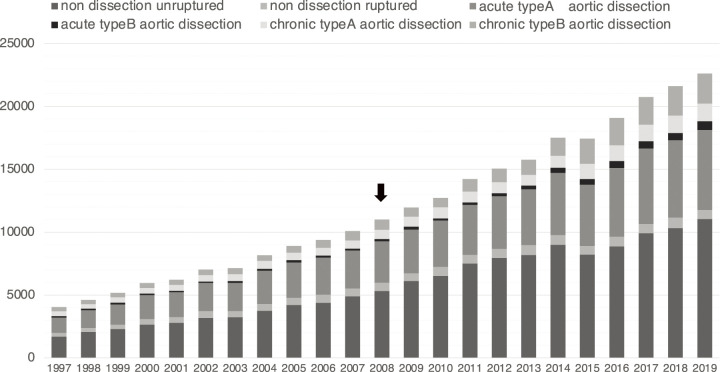
The number of aortic surgery according to disease in Japan.

The JATS annual surgery surveys have shown that the number of aortic surgeries has been increasing constantly over the last two decades, with rates approximately doubling in each decade: 5167 cases in 1999, 11956 cases in 2009, and 22708 cases in 2019 (**Fig. 1**).^3,4)^ The JATS annual surgery survey changed the data collection method from a questionnaire to data conversion from the Japan Cardiovascular Surgery Database in 2015. Therefore, there was a small decline in aortic surgeries in 2015.

## TEVAR IN JAPAN

**Fig. 2:**
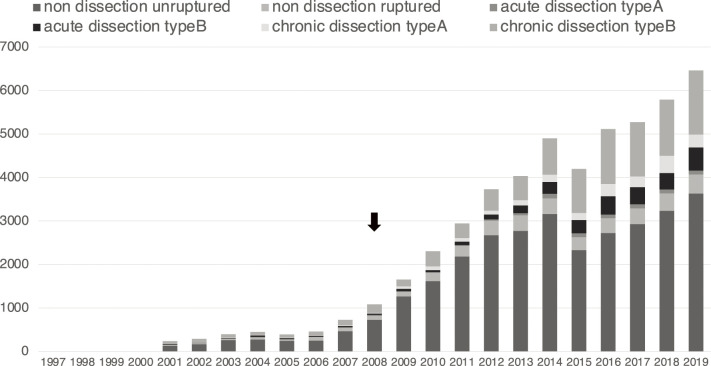
The number of TEVAR according to disease in Japan. The black arrow indicates the approval of commercially available stent grafts.

TEVAR was performed in a limited number of institutes in the early 2000s and was greatly enhanced by the approval of commercially available stent grafts in 2008. Its performance increased steadily thereafter, with 0 cases performed in 1999, 1658 in 2009, and 6461 in 2019. The ratio of TEVAR was 0% in 1999, and it was enhanced to 13.9% in 2009 and 28.5% in 2019, respectively (**Fig. 2**).

**Fig. 3:**
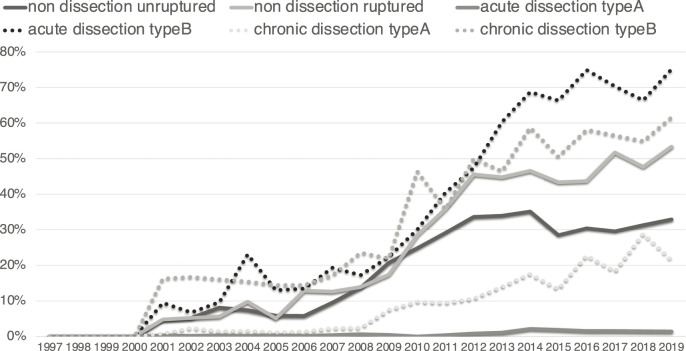
Ratio of TEVAR according to disease.

TEVAR is well indicated for diseases of the descending aorta, and its performance rate varies depending on the surgical range and aortic disease. In 2019, TEVAR was performed in 3633 cases of non-dissection unruptured aneurysms, 439 cases of non-dissection ruptured aneurysms, 89 cases of type A acute aortic dissection, 528 cases of type B acute aortic dissection, 302 cases of type A chronic aortic dissection, and 1470 cases of type B chronic aortic dissection. The ratio of TEVAR was high in type B acute aortic dissection (75%), type B chronic aortic dissection (62%), and non-dissection and ruptured aneurysms (53%) but low in type A acute aortic dissection (1%), type A chronic aortic dissection (21%), and non-dissection and non-ruptured aneurysms (33%) due to anatomical limitations (**Fig. 3**).

As TEVAR is less invasive than open surgery, its indication is expanding to the elderly population and high-risk patients with some morbidities. In non-dissection, non-ruptured aneurysms, TEVAR still showed an increasing trend even in 2019 and had not yet reached a plateau at that point. In type B acute and chronic aortic dissection, the number of TEVAR cases showed a dramatic increase owing to the recommendation of preemptive TEVAR for such pathophysiology in the guidelines,^5)^ and the rate of increase was even higher in 2019. In addition, with advances in stent-graft technology, the surgical indication for TEVAR is expanding to the aortic arch and thoracoabdominal region. The 30-day mortality rate for TEVAR was 2.3% in 2019 in non-dissecting, non-ruptured aneurysms. Although it is difficult to compare the surgical results between open surgery and TEVAR, the 30-day mortality rate of open surgery for the descending aorta, which was the same surgical range as TEVAR, was 4.7%. In addition, aortic arch replacement showed a 30-day mortality rate of 1.7%, which is superior to that of TEVAR. However, while indications for TEVAR are expanding to the aortic arch, surgical procedures should be selected based on its procedural outcomes.

**Fig. 4:**
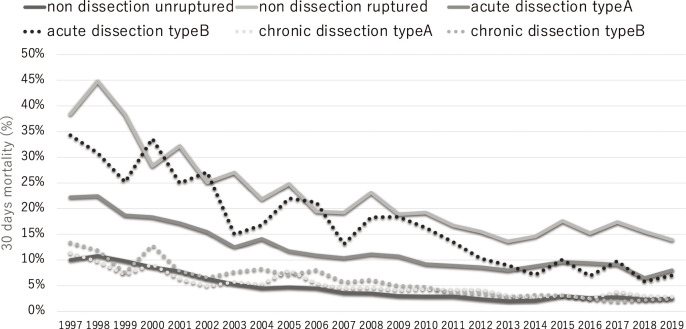
The 30-day mortality according to disease in patients undergoing aortic surgery.

TEVAR has greatly contributed to the improvement of surgical outcomes in non-dissection ruptured aneurysms and type B acute aortic dissection, which requires further improvement of surgical outcomes. TEVAR was performed in 53% of ruptured aneurysms, and the 30-day mortality rate improved to 13.9% in 2019 due a 30-day mortality rate of 12.5% in TEVAR for ruptured aneurysms. The effect of TEVAR was more remarkable in patients with acute type B aortic dissection, where the 30-day mortality rate was 5.7%, and the procedure was performed in 75% of cases. The overall 30-day mortality rate improved to 7.0% for all patients with type B acute aortic dissection in 2019 (**Fig. 4**).

## THE “S” CURVE THEORY IN TEVAR

The number of TEVAR still showed an increasing trend even in 2019 because the number of aortic surgeries has been increasing. However, the ratio of TEVAR plateaued in 2019, showing an “S” curve (**Fig. 3**). In contrast, the surgical outcomes have steadily been improving for the last two decades, even in high-risk surgeries, such as type A and B acute aortic dissection and non-dissection ruptured aneurysms. The spread of TEVAR has contributed to the improvement of these outcomes. It is also important to note the improvements in surgical outcomes of open surgery. The surgical outcomes for acute type A aortic dissection are mainly based on open surgery, as TEVAR cannot be indicated for this pathophysiology. However, it is internationally recognized that the surgical outcomes of acute type A aortic dissection in Japan are excellent.^6)^ Both improvements in surgical outcomes of open surgery and the spread of TEVAR have led to improved surgical outcomes of aortic surgery in Japan.

## CONCLUSIONS

The number of aortic surgeries has shown a continuously increasing tendency, doubling every 10 years over the last two decades, and surgical outcomes of aortic surgery have been improving as well. The spread of TEVAR using stent grafts greatly increased the number of aortic surgeries and played a significant role in improving surgical outcomes. Stent-graft technology has influenced the field of aortic surgery.

## AUTHOR INFORMATION

Akihiko Usui is a former Editor-in-Chief of *Surgical Case Reports*.
